# Dielectric Sphere Oligomers as Optical Nanoantenna for Circularly Polarized Light

**DOI:** 10.1021/acsphotonics.4c00761

**Published:** 2024-07-20

**Authors:** Shintaro Ogura, Hidemasa Negoro, Izzah Machfuudzoh, Zac Thollar, Tatsuki Hinamoto, F. Javier García de Abajo, Hiroshi Sugimoto, Minoru Fujii, Takumi Sannomiya

**Affiliations:** † Department of Materials Science and Engineering, School of Materials and Chemical Technology, Tokyo Institute of Technology, 4259 Nagatsuta, Midori-ku, Yokohama 226-8503, Japan; ‡ Department of Electrical and Electronic Engineering, Graduate School of Engineering, Kobe University, Kobe 657-8501, Japan; § ICFO-Institut de Ciencies Fotoniques, The Barcelona Institute of Science and Technology, Castelldefels, Barcelona 08860, Spain; ∥ ICREA-Institució Catalana de Recerca i Estudis Avançats, Passeig Lluís Companys 23, Barcelona 08010, Spain

**Keywords:** spherical resonator, Mie mode, cathodoluminescence, scanning transmission electron microscopy, circularly polarized light

## Abstract

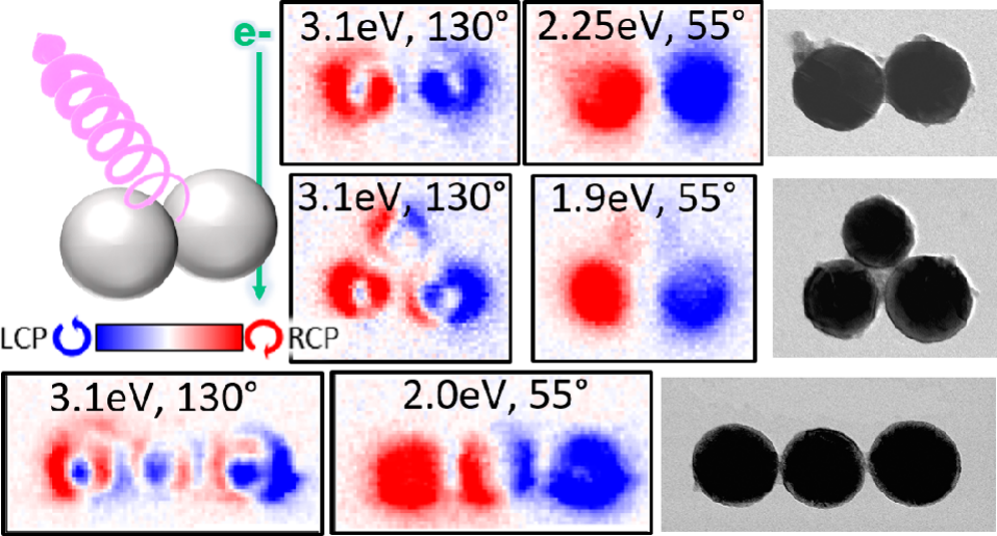

Control of circularly polarized light (CPL) is important for next-generation optical communications as well as for investigating the optical properties of materials. In this study, we explore dielectric-sphere oligomers for chiral nanoantenna applications, leveraging the cathodoluminescence (CL) technique, which employs accelerated free electrons for excitation and allows mapping the optical response on the nanoscale. For a certain particle-dimers configuration, one of the spheres becomes responsible for the left-handed circular polarization of the emitted light, while right-handed circular polarization is selectively yielded when the other sphere is excited by the electron beam. Similar patterns are also observed in trimers. These phenomena are understood in terms of optical coupling between the electric and magnetic modes hosted by the dielectric spheres. Our research not only expands the understanding of CPL generation mechanisms in dielectric-sphere oligomer antennas but also underscores the potential of such structures in optical applications. We further highlight the utility of CL as a powerful analytical tool for investigating the optical properties of nanoscale structures as well as the potential of electron beams for light generation with switchable CPL parities.

## Introduction

Encoding information in the polarization of light is swiftly becoming an alternative enabling technology for the transfer of optical information in classical and quantum communication schemes.^1,2^ In addition, circularly polarized light (CPL) could be useful for reading out material information as well as for robust signal transferring compared to linear polarization.^3−6^ For such applications, switchable CPL light sources are required, where an emitting material or an emitter-coupled antenna should respond equally to light with right-handed and left-handed circular polarizations (RCP and LCP). Since chiral structures respond differently to CPL of different parity, the use of symmetric, achiral structures such as nanoantennas and waveguides has been extensively explored.^7−9^ Even the ultimately symmetric structure, a sphere, can work as a CPL nanoantenna whose CPL parity is controllable by the use of an electron beam (e-beam) through the extrinsic chirality (i.e., breaking the symmetry of the object by the detection and excitation geometry^10−12^). Such sphere antennas have omnidirectional responses and, for example, can be placed in liquids, where the orientation of the antennas is random.^13^ In this configuration, the chirality of the system is controlled extrinsically by the excitation and detection directions. The CPL functionalities of spherical nanoantennas are based on the Mie resonances supported by the spherical structure. Compared to plasmonic spheres, dielectric spheres made of high-index materials are considered suitable building blocks for low-loss nanophotonic applications, such as linear arrays and metasurfaces with periodic structures that operate as waveguides and can benefit from topological features.^14,15^

Dielectric nanoantennas consisting of a finite number of particles are also applied to lasing, sensing, and nonlinear optics applications, which are made possible by the availability and controllability of both magnetic and electric resonances with comparably low losses.^16^ However, for CPL applications, nanoantennas consisting of multiple identical dielectric spheres or oligomers (e.g., dimers, trimers, etc.) have scarcely been investigated, although there are a number of studies addressing the optical coupling of dielectric spheres.^17,18^ We also note that the experimental characterization of such oligomers has so far been done by acquiring optical scattering spectra, which are hard to interpret and heavily depend on experiment-simulation comparisons.^17,19,20^

**Figure 1:**
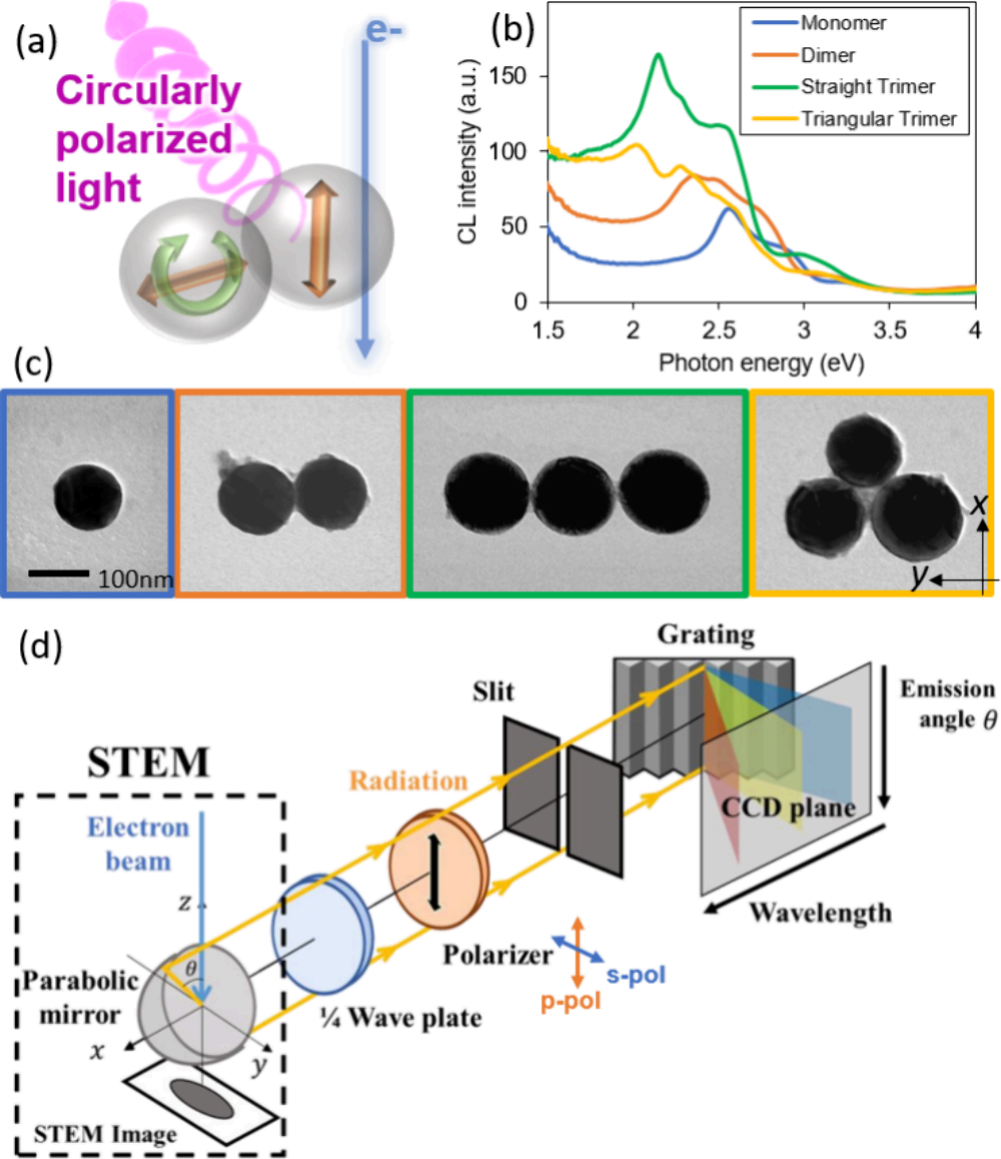
Chiral light emission in dielectric sphere oligomers and their characterization through four-dimensional (4D) STEM-CL. (a) Concept of CPL generation upon electron beam (e-beam) excitation of a dielectric sphere dimer. (b,c) CL spectra (b) and STEM-BF (c) images of the investigated silicon sphere oligomers, namely, a monomer, a dimer, and trimers with linear and triangular configurations (from left to right). (d) Illustration of our 4D STEM-CL system with the angular- and wavelength-(energy)-resolved data stored for each e-beam position.

In this study, we experimentally investigate Si sphere oligomer nanoantennas for the generation of CPL using cathodoluminescence (CL), where the accelerated free electrons serve as point-like excitation sources on the lateral plane at designate (*x*_*e*_, *y*_*e*_) positions with its excitation phase varying along the e-beam path *z*, effectively representing a line dipole of polarizability ∝*e*^*i*(ω/*v*)*z*^**z**^21,22^ (see Figure 1). The e-beam excitation approach is advantageous for generating CPL with fast switching of polarization.^10^ The CL method is also a powerful tool for analyzing the optical properties of dielectric nanoantennas, as it can visualize the optical near field with spatial resolution far beyond the diffraction limit of light using the detection angle and polarization selectivity to take advantage of the extrinsic chirality.^23,24^ For the simplest oligomer antennas, we choose dimers and trimers (see Figure 1c). Under specific conditions, we observe that excitation on one sphere at any place yields left-handed circular polarization (LCP) in a dimer, while excitation on the other sphere always yields right-handed circular polarization (RCP) – an effect that we attribute to coupling to two electric dipoles with different phases. Similar patterns are also observed for trimers in both linear and triangular conformations.

## Methods

### Sample Fabrication

Silicon sphere oligomers were fabricated through template-assisted self-assembly of Si spheres.^25^ The Si spheres were first grown in a SiO_2_ matrix by thermal disproportionation of silicon monoxide lumps at 1475 °C, and subsequently extracted from the matrix by HF etching. Size-separated Si spheres were then obtained by a density gradient centrifugation process. In this work, Si spheres with diameters of around 120 nm were used. The size-separated Si spheres were aligned in templates (i.e., 200–1000 nm-width grooves fabricated on a polymer surface) through a template-assisted self-assembly process. To transfer aligned Si spheres to a TEM mesh, ethyl acetate solution of cellulose acetate butyrate (CAB) was deposited on a template and dried in air to form a CAB film. The film was detached from the template in water and placed on a C-coated Cu mesh. Dissolution of CAB by ethyl acetate resulted in the transfer of Si sphere arrays onto the mesh.

### Cathodoluminescence Measurements

Representative spectra and the bright-field STEM images of the investigated Si sphere antennas are shown in Figure 1b and c. For the CL measurement, a modified STEM (2100F, JEOL Japan) instrument equipped with a Schottky-type field emission gun and an aberration corrector is operated at 80 kV acceleration with an e-beam current of 1 nA.^26^ The parabolic mirror situated at the sample position collimates the light emission from the sample. Only the ϕ = 0° azimuthal angle component of the emission is selected by a slit mask while components for polar angles in the θ = 0–180° range are detected in a CCD camera to obtain 2D information on the θ angle and wavelength at each e-beam position (Figure 1).^10^ Thus, when performing an e-beam scan, 4D data sets are obtained. A polarizer and a phase plate are inserted in the optical path for polarimetric analysis. The dispersion of the phase plate and the phase shift due to reflection by the mirror are corrected. A lateral shift and a shear of the image due to sample drift during the mapping were corrected for the RCP and LCP maps to plot the subtraction CPL maps (RCP-LCP).

### Simulations

To analyze the coupling to and among modes of Si spheres, we performed numerical simulations based on multiple scattering of the fields induced on each sphere both by the passage of the e-beam and by the interaction among different particles. More precisely, we decomposed the field around each particle in spherical waves and followed a multiple elastic scattering of multipole expansions (MESME) approach to self-consistently determine the spherical wave amplitudes.^27,28^ We followed an iterative procedure that converged after 20 iterations. To map the field distribution for visualization of the dipole rotation, we utilized a multiple multipole program (MMP) with an e-beam excitation, which also allowed extracting a certain multipole mode.^29,30^ Like in experiment, the e-beam energy was set to 80 keV for all the simulations.

## Results and Discussion

### Monomer

**Figure 2:**
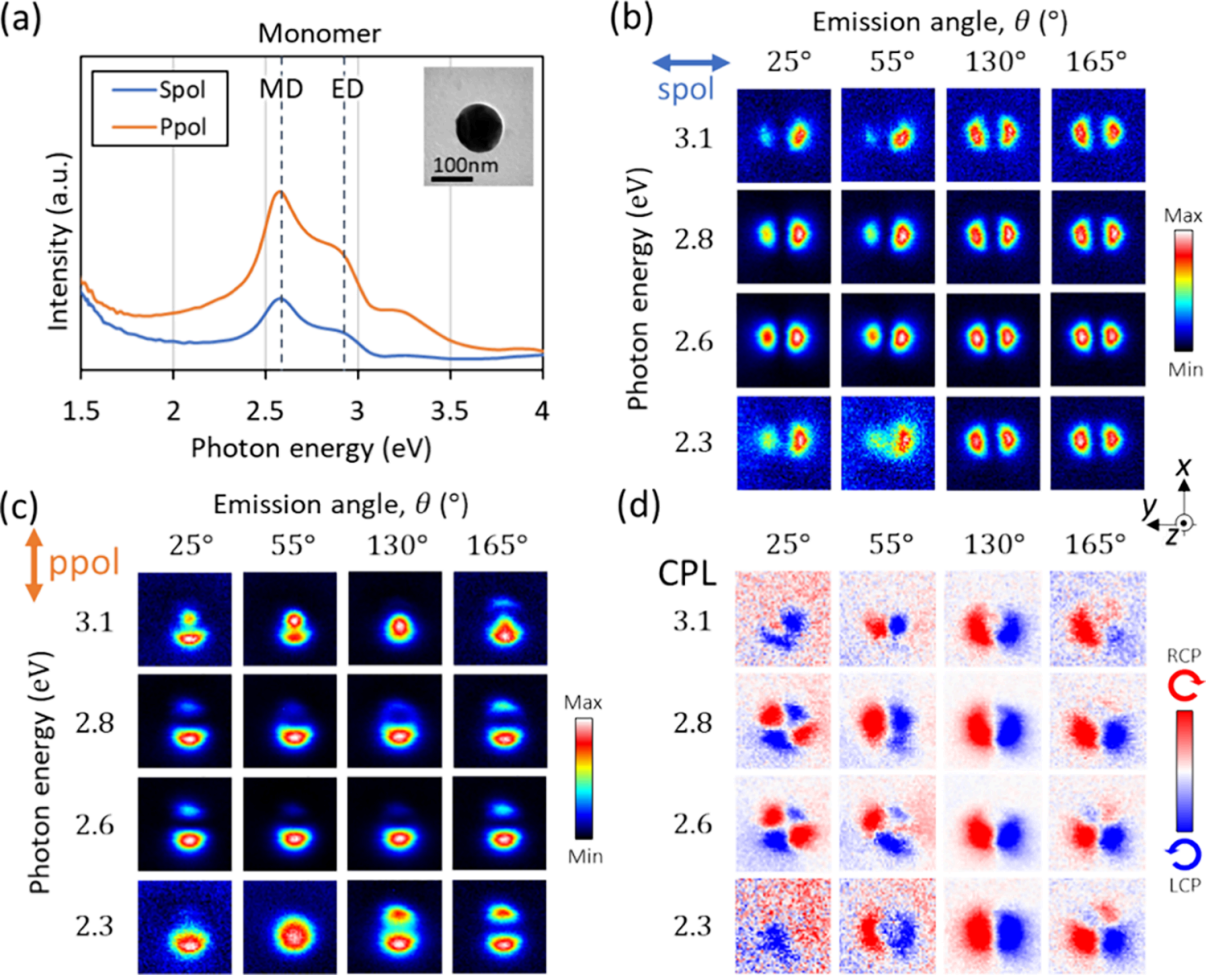
CL measurements for a single Si sphere. (a) CL spectra integrated over all detection angles (θ = 0–180°) and e-beam positions on the entire sphere. Emission with *s* and *p* polarization corresponds to the optical electric fields polarized along the *y* axis and on the *x*-*z* plane, respectively. (b-d) CL mapping collected at different detection angles and energies for (b) *s* polarization, (c) *p*1 polarization, and (d) CPL emission.

We first investigate an isolated individual Si sphere (monomer) as a reference for the oligomers in the next sections. The CL spectrum of the monomer (Figure 2a) shows clear magnetic dipole (MD) and electric dipole (ED) features as well as higher order modes at the higher energies. These two dipole modes are also visible in the *s*- and *p*-polarization CL mappings, as shown in Figure 2b and c. In the CL-CPL mapping in Figure 2d, we observe RCP and LCP emission on each side of the sphere along the *y* axis for a detection angle around the *x* axis (θ = 130°), showing a pattern with *two lobes* with opposite CPL parities, which indicates ED mode rotation.^10^ Also, a *four-lobe* pattern with alternating CPL parities is observed close to upward emission (θ = 25°, 2.6–2.8 eV), which results from the interference of MD and ED modes, as discussed in the previous study.^10^ Such an interference of different energy modes takes place when their resonances are broad and spectrally overlapping.^23,31^

### Dimer

**Figure 3:**
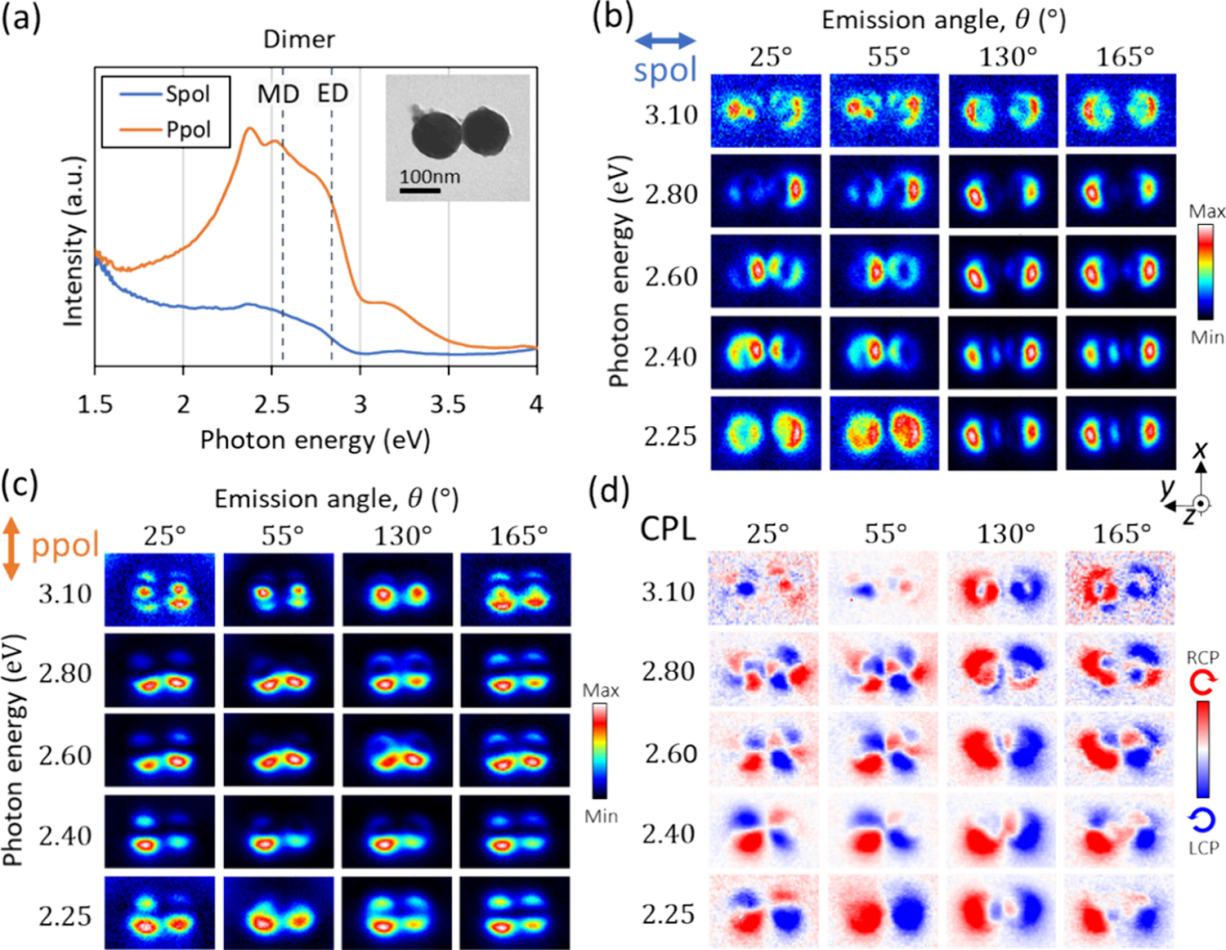
CL measurements for a Si-sphere dimer. (a) CL spectra integrated over all detection angles (θ = 0–180°) and e-beam positions on the entire dimer. Emission with *s* and *p* polarization corresponds to the electric field polarized along the *y* axis and on the *x*-*z* plane, respectively. (b-d) CL mapping collected at different detection angles and energies for (b) *s* polarization, (c) *p* polarization, and (d) CPL emission.

Results for a dimer are summarized in Figure 3. Compared to the monomer, the dimer displays more features in the CL spectrum at lower energies, as shown in Figure 3a (see also Figure 1b for comparison). It should be noted that the sphere size in the dimer is slightly larger than the investigated monomer of Figure 2 according to the STEM-BF image in the inset of Figure 3a, which can also be recognized as the MD-related feature around 2.5 eV, which is discussed later together with simulations, appearing slightly at a lower energy than the monomer. The main contribution to the broad spectral features around 1.5–2.5 eV is related to coupled ED-ED mode.^32,33^ Also, ED modes tend to have broad spectral features at low energies compared to MD modes, and the latter have sharper resonances. Considering the orientation of the dimer aligned along the *y* axis, the coupled ED-ED mode produces the lowest energy features in the *s*-polarized field (electric field along the *y* axis) when excited at the edges along the *y* axis, while the MD-MD mode with this excitation contributes to the *p*-polarized field (electric field on the *x*-*z* plane) when excited at the edges along the *x* axis.

The *s*-polarized field pattern in Figure 3b clearly shows such coupled ED-ED mode at the lower energies (2.25–2.40 eV) for angles in the 130–165° range, with strong fields located near the outer edges of the spheres. The inner edge field of this mode is not sensitively detected by CL because only the *z* component of the electric field is monitored (i.e., coupled to) by the e-beam.^34^ (see SI for schematic illustrations) Similar features of highlighted edges are also found at the highest energies, which we attribute to electric quadrupoles (EQs).^23^ The strong inner-edge intensities that are observable in the upward emission (25–55°) correspond to the features associated with the MD modes having the magnetic poles aligned on the *x-z* plane. The outer edge features are dimmed at these angles because of the cancellation of the fields of the MD and ED modes, in the same manner as in the so-called Kerker effect.^35^ (see SI) The *p*-polarization CL map (Figure 3c) shows similar features to the MD or ED modes of an individual sphere, especially at low energies.

**Figure 4:**
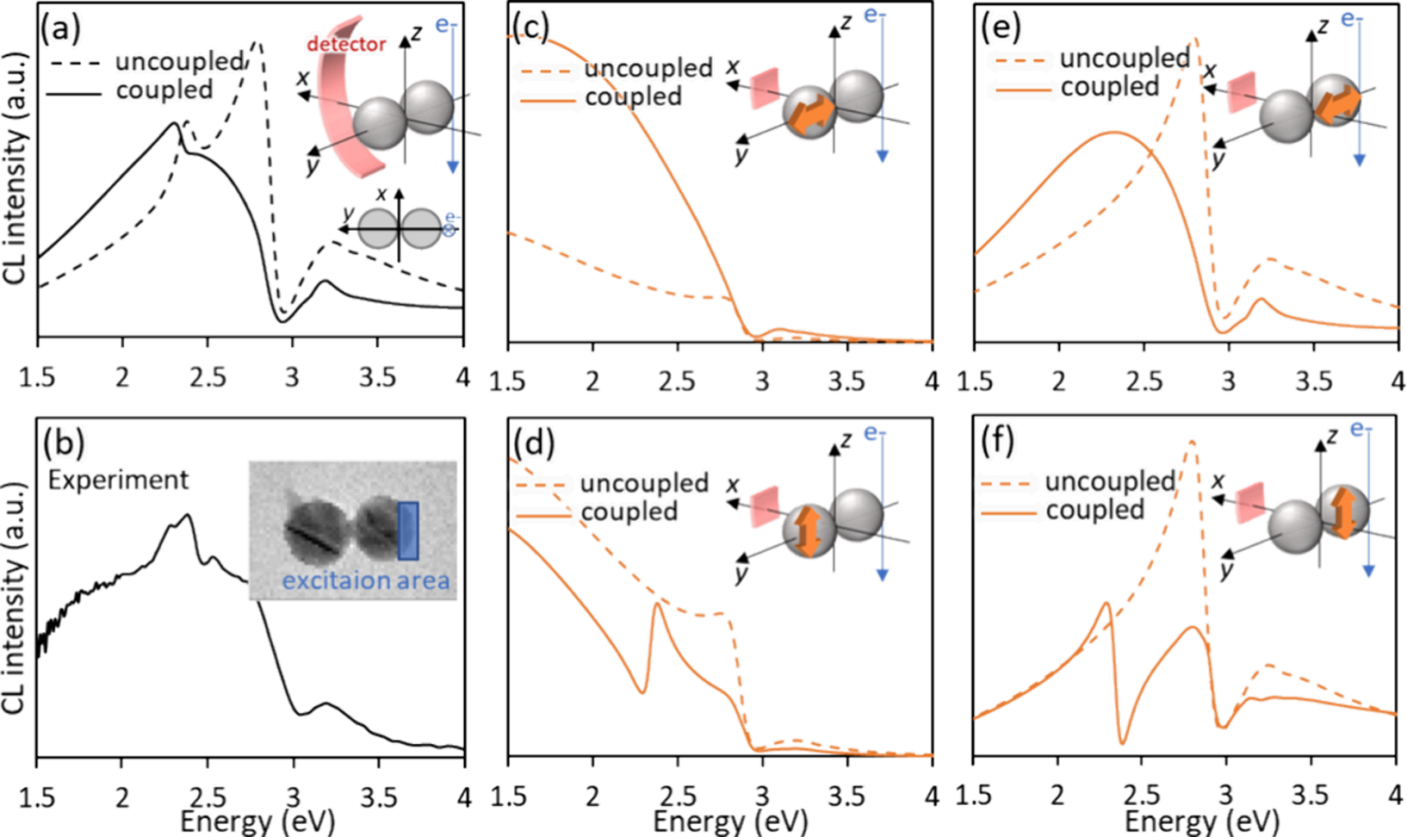
Experiment-simulation comparison for coupled electric dipoles in a Si dimer. Simulations are performed using MESME for 120 nm Si spheres separated by 5 nm gaps without polarization. The e-beam travels along the *z* axis and passes 7.5 nm away from the sphere surface on the negative *y* axis. (a,b) θ-integrated CL spectra of (a) the simulated emission intensity and (b) the corresponding experimental measurements. (c-f) Simulated spectra from a specific electric-dipole component for coupled and uncoupled dimers with a detection angle θ = 90°. The orientation and position of the extracted electric dipole are schematically illustrated in each panel. The pink rectangular shape in the inset represents the position of the detector.

The coupling effect of the dimer modes can be unraveled using the MESME method, which allows calculating the contribution of a given mode for coupled and uncoupled states.^27,28^ We first confirm that the CL spectrum is neatly reproduced in the MESME simulation, as shown in Figure 4a,b. The simulation is performed for two 120 nm Si spheres separated by a 5 nm gap and with the e-beam excitation at the *y* negative edge of the dimer, as illustrated in Figure 4. We verified the gap size in the STEM image and also confirmed that the gap distance does not significantly affect the CL spectral features (see SI). Basically, the uncoupled spectra (dotted lines) correspond to those of individual spheres. To see the coupling effect of EDs, we extract spectra of only a certain ED component at a detection angle θ = 90° (Figure 4c-f). The *y*-axis-oriented EDs (Figure 4c,e) show broad features of the coupled ED-ED mode. In contrast the *z*-oriented-EDs (Figure 4d,f) show MD features at an energy around 2.4 eV, although only the ED component is extracted. This indicates that *z*-oriented EDs are coupled to MDs. We also note that the contribution of the *y*-axis-oriented ED of the sphere away from the e-beam (Figure 4c) is strongly enhanced at lower energies below 2.7 eV due to ED-ED coupling. We note that the ED components dominate the CL intensity in this configuration (see SI).

Knowing that mode coupling significantly influences the optical properties, we now examine the CPL mapping results, as shown in Figure 3d. At a high photon energy (3.1 eV), higher-order modes as well as their hybridized combinations give rise to complex field patterns both in the CPL and linear polarization maps. At energies in the 2.6–2.8 eV range and angles of 25–55°, a four-lobed feature in each sphere is observable in the CPL map (Figure 3d), which is similar to the individual sphere (see Figure 2). At lower energies, CPL generation involves the aforementioned coupled modes. At energy of 2.25–2.40 eV and angles of 25°, a four-lobe CPL pattern of the entire dimer (two lobes for each sphere) is found. This feature can be attributed to the interference of the ED and MD modes from each sphere, which are not coupled, similar to the interference of ED and MD modes in the individual sphere,^10^ thus producing similar patterns in the monomer and the dimer.

**Figure 5:**
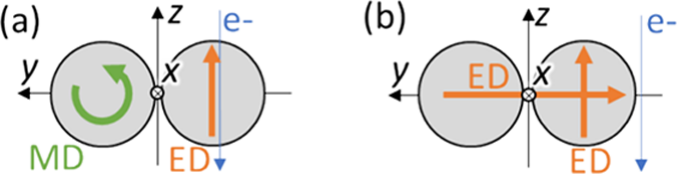
Illustrations of two interfering modes to generate CPL emission from a Si dimer. The orange and green arrows represent the electric field of ED and MD modes, respectively. The blue arrow depicts the e-beam. (a) ED-MD coupled with the *z* component of the ED responsible for the *p*-polarization electric field (along *z*) and the MD for *s* polarization. (b) Interference of the *z* component of the ED mode and the horizontally coupled ED, which contribute to *p*- and *s*-polarization signals, respectively. While the configuration of panel (a) gives no MD radiation toward the *x*-axis direction, a slight elevation of the detection (radiation) angle produces a contribution of the MD component with *s* polarization (*y* component of the electric field).

For a detection angle of 55°, closer to the horizontal *x-y* plane at an energy of 2.25 eV, the CPL pattern shows only RCP contrast on the entire left sphere and only LCP on the entire right sphere. This interesting CPL distribution can be attributed to the coupled ED-MD mode, as described above in Figure 4d,f, and also to interference of the coupled horizontal ED-ED mode along the *y* axis and the perpendicular ED mode along *z* axis. In the former case of ED-MD coupling, the perpendicular *z*axis-oriented ED mode is excited in the sphere on which the e-beam hits, and the coupled MD mode in the neighboring sphere should be polarized along the *x* axis with the rotating electric field around the *x* axis direction (see Figure 5a for the illustration). This coupled MD mode has no emission toward the *x* axis (θ = 90°). However, a slight elevation of the angle θ permits detecting the radiation emanating from this *x*-polarized MD mode, with a dominant contribution in the *s*-polarization component (*y* component of the electric field). In this way, with a certain phase difference between the two modes, the coupled ED-MD mode can generate CPL emission with flipping signs (because of the flipping sign of the MD mode) by selecting the sphere that the e-beam is impacting.

In the latter scenario, with interfering EDs, the situation is similar to the individual sphere:^10^ The horizontal ED (i.e., the ED-ED coupled mode) contributes to the *s*-polarization component, while the perpendicular ED contributes to the *p*-polarization component, as illustrated in Figure 5b. With a certain phase difference, CPL is radiated along the *x* axis, and the sign of the CPL is flipped depending on whether the left or right sphere is excited, which flips the phase of the horizontal dipole. This mechanism corresponds to a rotating ED in the entire system. To describe this situation, we show the field mapping in the SI along with the extracted ED components using MMP simulations.^29^

At an energy of 3.1 eV and an angle of 130° in the CPL mapping in Figure 3d, two-color features with RCP in the entire left sphere and LCP in the entire right sphere are found, which are similar to the electric dipole rotation discussed above. However, in this case, each sphere displays a *donut* pattern with the center of the sphere having no signal. For the individual sphere at this energy (3.1 eV) and angle (130°), the *p*-polarization component is mostly contributed by the electric quadrupole mode with an azimuthal number *m* = 0 mode, as shown in Figures 2c and 3c.^23,36^ The *s*-polarization component seems to originate from the coupled electric dipole of the radial second mode (*n* = 2, with *n* being the radial order), considering the mapping patterns with intensities both inside and outside the sphere edges in Figure 3d as well as the radiation direction and polarization.^36^ Consequently, this donut CPL pattern can be understood as the result of interference between the *m* = 0 electric quadrupole for the *p*-polarization component and the second-order electric dipole (*n* = 2) for the *s*-polarization component.

### Linear Trimer

**Figure 6:**
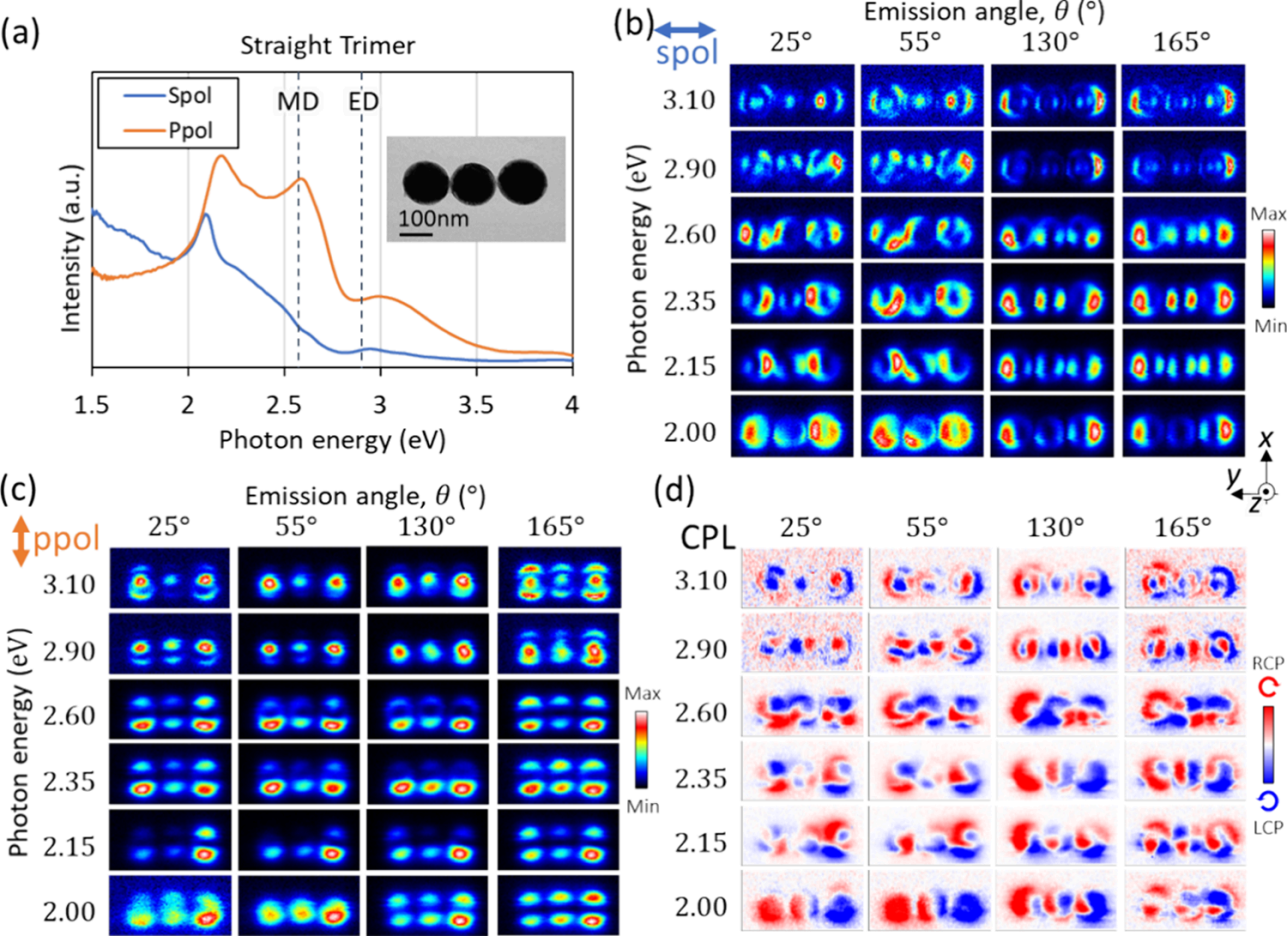
CL measurements for a Si-sphere linear trimer oriented along the *y* axis. (a) CL spectra integrated over all detection angles (θ = 0–180°) and e-beam positions on the entire trimer. Emission with *s* and *p* polarization corresponds, respectively, to optical electric fields polarized along the *y* axis and on the *x*-*z* plane. (b-d) CL mapping results acquired at different detection angles and energies for (b) *s* polarization, (c) *p* polarization, and (d) CPL.

We investigate a linear trimer aligned along the *y* axis. This trimer with a linear configuration exhibits spectral features at even lower energies than the dimer discussed in the previous section, as shown in Figure 6a (see also a direct comparison in Figure 1b). These low-energy features are related to coupled modes extending along the chain axis.^17^ The CPL mapping in Figure 6d shows patterns of connected features of the coupled modes at the lowest energies, similarly to the dimer. The CPL map patterns are not like those of individual spheres, but instead, they exhibit coupled-mode features even at the highest energies, characterized by complex intensity distributions that indeed corroborate the coupled nature of such higher-order modes. In the *s*-polarization maps at the corresponding energy of 2.0 eV in Figure 6b, a Kerker-like interference effect of the coupled ED and individual MD produces the bright edges only in the downward emission (θ > 90°), similarly to the dimer configuration. This type of interference is not observed in the upward emission (θ < 90°) (see Supporting Information). The *p*-polarization maps in Figure 6c do not show complex patterns compared to *s* polarization because the *z* component of the electric field amplitude (i.e., the one probed through CL by electrons moving along *z*) of the magnetically coupled modes do not significantly differ from the individual magnetic modes. The ED-MD coupling, similarly to the dimer, is also responsible for the spectral features, which are investigated through MESME simulations in the SI.

The CPL maps collected at an energy of 2.0 eV and angles of 25° and 55° (Figure 6d), show RCP emission on the left half and LCP on the right half of the structure. This indicates that the horizontally coupled electric-dipole mode interferes with the *z* component of the dipole in the excited sphere to generate RCP or LCP emission, depending on the position of the e-beam in a similar manner as discussed in Figure 4. A four-lobe-like CPL pattern from each sphere, originating from the interfering in-plane ED and MD modes, is also observed at an angle of 25° and an energy of 2.35 eV, although the pattern is not very symmetric due to imperfections in the structure. This energy is even lower than that for the dimer (and the monomer), indicating the lowered mode energies of the coupled modes. At the energy of 3.1 eV, no donut shape pattern is found for this trimer, in contrast to the dimer, although *m* = 0 quadrupoles are apparent in the *p*-polarization mapping in Figure 4d. This can be interpreted as the result of phase mismatch between the second-order electric dipole and the first-order mode, which are accountable for the generation of donut patterns in the sphere.

### Triangular Trimer

**Figure 7:**
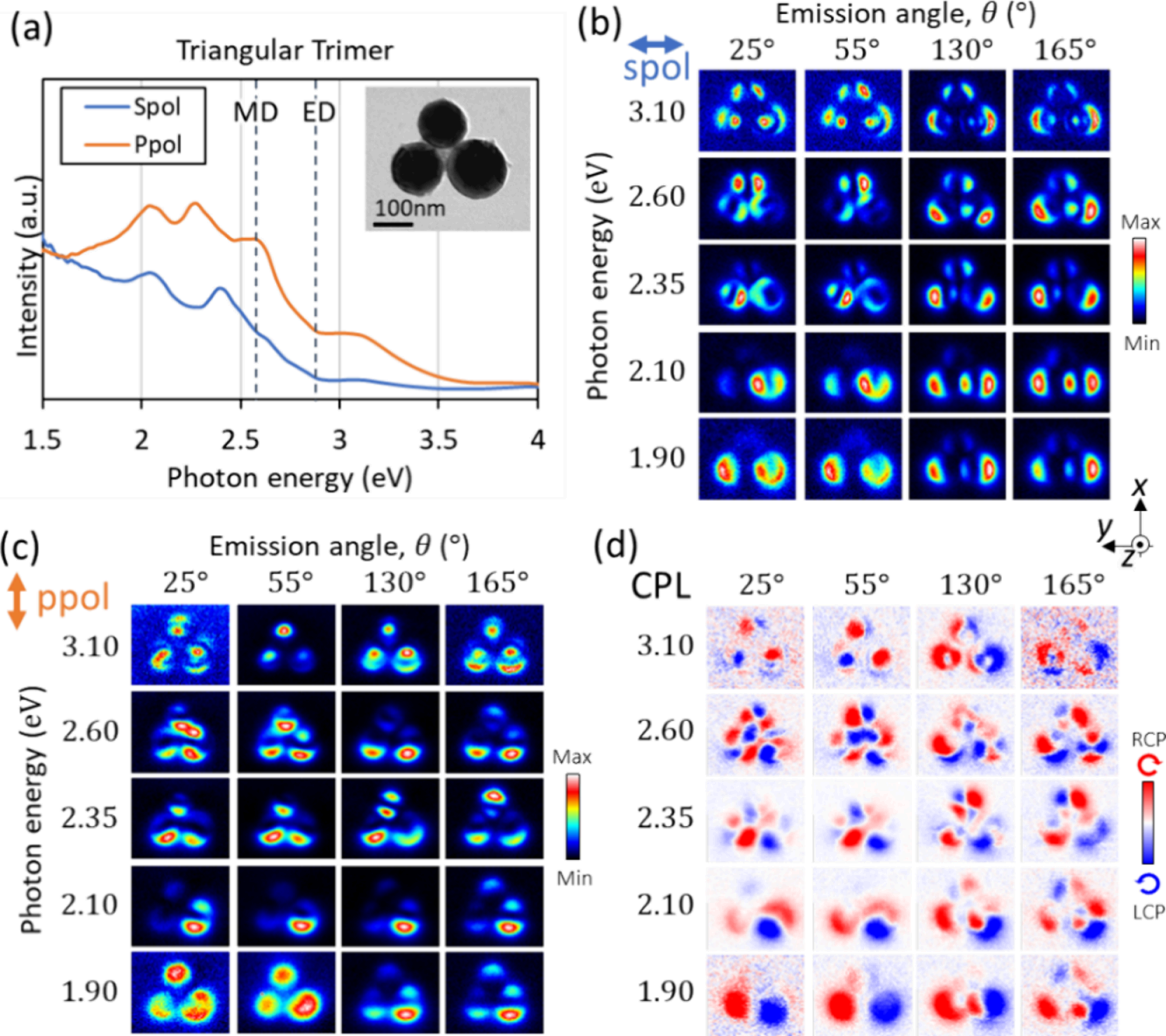
CL measurements for a Si-sphere trimer in a triangular conformation. (a) CL spectra integrated over all detection angles (θ = 0–180°) and e-beam positions on the entire trimer. Emission with *s*- and *p*-polarized light corresponds, respectively, to optical electric fields oriented along the *y* axis and on the *x*-*z* plane. (b-d) CL mapping results acquired at different detection angles and energies for (b) *s* polarization, (c) *p* polarization, and (d) CPL emission.

Trimers can be arranged in different conformations (i.e., not only straight, like in a dimer). Here we show results for a trimer in a triangular configuration. As shown in Figure 7a, the spectral intensity extends to lower energies, similar to the linear trimer. In the CPL map at an energy of 1.9 eV and an angle of 55° in Figure 7d, RCP and LCP light emissions are nicely separated on the left and right halves of the structure, which is like in a dimer for the bottom two (Figure 3d at 2.25 eV, 55°) and in an individual sphere for the top one (Figure 2d at 55° and 135°). The corresponding *s*-polarization maps show an electric dipole-like polarization in the horizontal direction (Figure 7b), corresponding to the so-called E’ mode, with strong dipole polarization in the same direction for the bottom two particles and a weak one for the top particle.^37,38^ In the *p*-polarization maps at the corresponding energy (1.9 eV) and angle (55°) in Figure 7c, the intensity is distributed on the entire spheres, indicating that the excitation of the out-of-plane electric dipole (polarized along the *z* axis) is dominant. Thus, the CPL generation with the neat separation of RCP and LCP on the right and left sides of the trimer (Figure 7d, 1.9 eV, 55°) can be qualitatively understood as the sum of emissions from the dimer and the monomer on top: the *z* component of the electric dipole of each sphere is responsible for the *p*-polarization component, and the horizontally coupled electric mode as well as the possible coupled ED-MD mode contributes to the *s*-polarization component.

For this triangular trimer, we observe a four-lobe like pattern on each sphere in the CPL map (Figure 7d) at an energy of 2.60 eV and an angle of 25°. This pattern is however connected to neighbor patterns and its energy is similar to the dimer rather than to the linear trimer. At an energy of 3.1 eV, a donut shape appears from the bottom two spheres for the angle of 130°, similar to the dimer. The top sphere shows a feature analogous to the second order ED of the single sphere.^10^

## Conclusions

We have demonstrated the generation of CPL light from dielectric sphere oligomers (namely, a dimer and trimers in different configurations) using e-beam excitation. The CL mapping results show various CPL patterns depending on the photon detection angles and energies. Under specific conditions, one of the particles produces only one parity of the emitted CPL, which is switched by moving the e-beam onto another particle. Our numerical simulations indicate that this peculiar CPL generation tunability is enabled by optical coupling among the particles. Such controllable CPL emission properties of dielectric sphere oligomers provide us with additional freedom in engineering CPL nanoantennas and are potentially useful for CPL sources based on e-beams. With a suitable design of the particle arrangement, control over polarization of the generated light could be achieved with a less precise location of the e-beam excitation spot, thus adding robustness to the generation scheme.

## Supporting Information Available

Kerker effect for dimers, MMP simulations of the rotating ED in a dimer, and MESME simulation of a linear trimer, gap-size evaluation, contribution of ED modes in a dimer (PDF)
