# Supplementary material for: Dielectric Sphere Oligomers as Optical Nanoantenna for Circularly Polarized Light
Source: ACS Photonics. 2024 Jul 20;11(8):3323–30. doi: 10.1021/acsphotonics.4c00761 (PMC11342412; doi:10.1021/acsphotonics.4c00761)
Supplement: Supplementary file 1 — ph4c00761_si_001.pdf [file ph4c00761_si_001.pdf]

Supporting Information for

Dielectric sphere oligomer as optical nanoantenna for circularly polarized light

Shintaro Ogura[1], *Hidemasa Negoro*[2], *Izzah Machfuudzoh*[1], *Zac Thollar*[1], *Tatsuki Hinamoto*[2], *F. Javier García de Abajo*[3,4], *Hiroshi Sugimoto*[2], *Minoru Fujii*[2], *Takumi Sannomiya*[1*]

AUTHOR ADDRESS

[1] Department of Materials Science and Engineering, School of Materials and Chemical Technology, Tokyo Institute of Technology, 4259 Nagatsuta, Midori-ku, Yokohama 226-8503 Japan

[2] Department of Electrical and Electronic Engineering, Graduate School of Engineering, Kobe University, Kobe 657-8501, Japan

[3] ICFO-Institut de Ciencies Fotoniques, The Barcelona Institute of Science and Technology, 08860 Castelldefels (Barcelona), Spain

[4] ICREA-Institució Catalana de Recerca i Estudis Avancats, Passeig Lluís Companys 23, 08010 Barcelona, Spain

*sannomiya.t.aa@m.titech.ac.jp

S1. "Kerker effect" for dimers

Figure S1 illustrates the Kerker effect for a dimer with the MD modes and the coupled EDs.

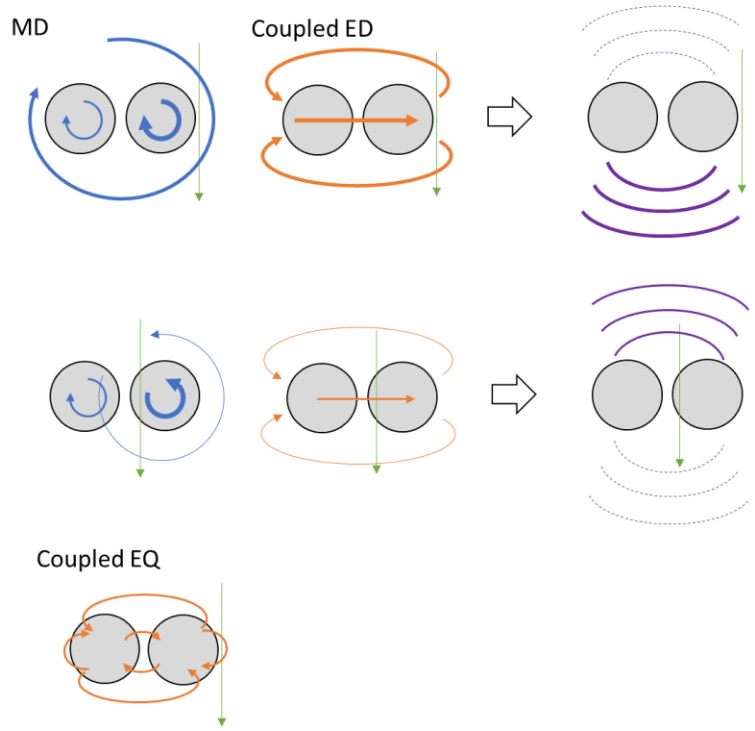

Figure S1. Illustration of coupled modes and their interference. The green arrow indicates the electron beam direction. The blue and orange arrows show electric fields originated from the magnetic and electric modes, respectively. In the interference patterns (rightmost column), the interfered wave fronts are illustrated by violet lines, showing Kerker-type effects.

S2. MMP simulations of the rotating ED in a dimer

To visualize the ED rotation, an electromagnetic simulation using a multiple multipole program (MMP) is performed for a dimer of 120 nm Si spheres separated by 5 nm at a photon energy of 2.25 eV. To visualize how the fields are distributed and generate CPL emission, the cross-sectional field on the *y*-*z* plane is plotted when the electron beam (e-beam) moves along the *z* axis and crosses the *y* axis 7.5 nm away from the surface of the sphere. Figure S2 shows the simulation results for the electric-field distribution on the *y*-*z* plane at different phases to visualize the time evolution at the selected energy of 2.25 eV. The electric field in Figure S2a shows a rotating field around the dimer on the *y*-*z* plane, clearly indicating CPL generation toward the *x* axis. We also extract multipole components to understand the field rotation, which is made possible by the multipole-based simulation. Since a dominant contribution of the (coupled) ED is expected at this energy, we plot the electric field of the extracted ED component in Figure S2b. While the right sphere close to the e-beam produces a strong *z*-dipole component, the left sphere shows mostly a *y*-dipole component, which is more clearly visible in the illustration of the dipoles in Figure S2c. This phase difference of the *y* and *z* components of the electric dipole is responsible for CPL generation, where the *y* component is essentially the coupled dipole mode. In the *all-field* map of Figure S2a, the contribution of the MD is also observable, showing a rotating electric field within the sphere, which however does not give rise to emission along the *x* axis.

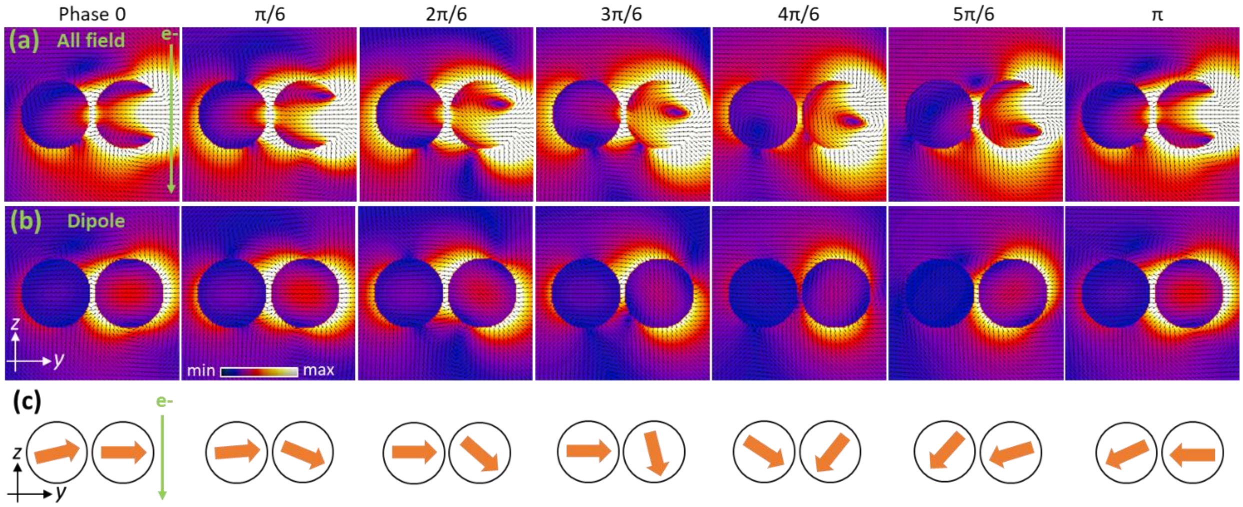

Figure S2. Simulated electric field maps for a dimer comprised of 120 nm Si spheres at a photon energy of 2.25 eV. The spheres are separated by 5 nm gaps. Like in experiment, the dimer is aligned along the *y* axis. We consider an electron beam with 80 kV acceleration voltage impacting the *y* axis at a distance of 7.5 nm from the right sphere surface. The electron travels along the negative *z* direction, as illustrated in the leftmost image. (a) Electric-field maps on the *y*-*z* plane at different phases with all multipolar components (spherical harmonics up to an order of 15) included. (b) Corresponding electric field maps extracted for only the electric-dipole component of each sphere. The small dark arrows superimposed on the field maps show the direction of the electric field. (c) Schematic illustration of the electric-dipole direction (orange arrows) of each sphere at the corresponding phases.

S3. MESME simulation of a linear trimer

The results of MESME simulations for a linear Si trimer are shown in Figure S3. The electric dipole components of each sphere are extracted for the coupled and uncoupled states.

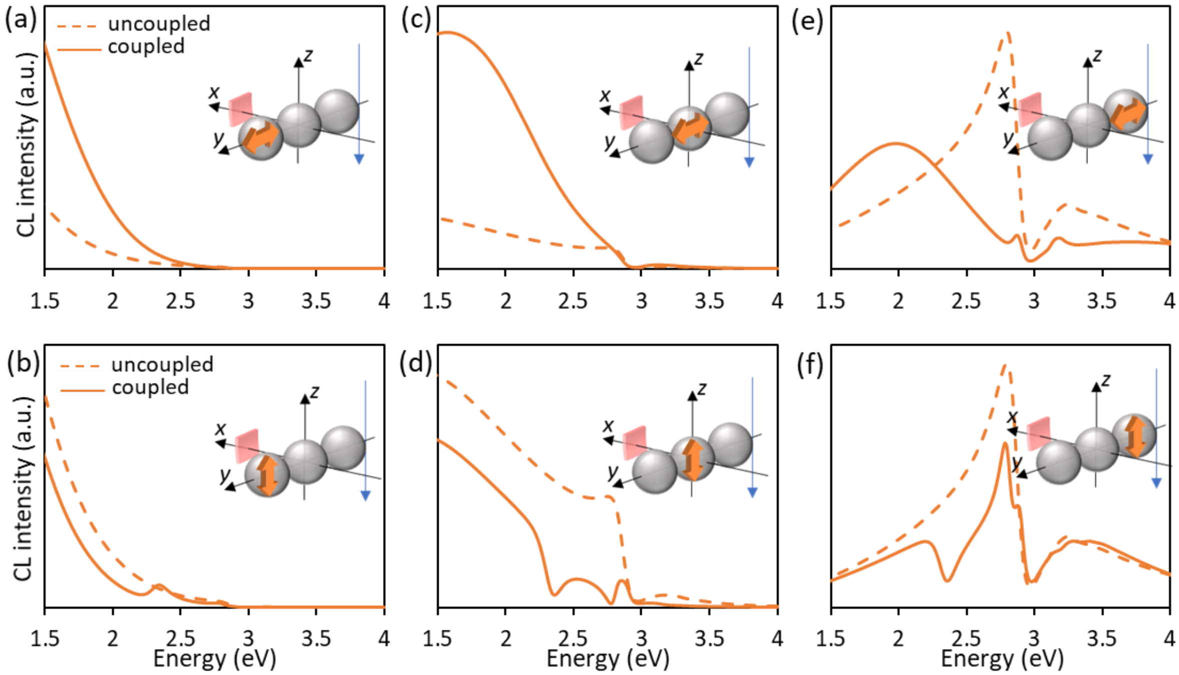

Figure S3. MESME simulations of coupled electric dipoles in a linear trimer consisting of 120 nm Si spheres separated by 5 nm gaps. The electron beam travels along the *z* axis and is incident 7.5 nm away from the surface of the sphere located at the *y*-axis negative end, as schematically illustrated in each inset. The pink rectangular shape in the inset represents the position of the detector. (a-f) Simulated CL spectra from a specific electric dipole component for coupled and uncoupled dimers with a detection angle $\theta = 90°$. The orientation and position of the extracted electric dipole are schematically illustrated by an orange arrow in each panel.

S4. Gap-size evaluation

Figure S4 shows magnified STEM images of the gaps between Si spheres in a trimer. At the gap, there remains some light-element substance, which is probably residual cellulose acetate butyrate (CAB) used in the fabrication process to transfer the arrayed spheres from the template to the TEM support membrane. The gap size can be reasonably estimated to be around 5 nm. The effective distance could also change depending on the refractive index of the residual CAB, which is ~ 1.48. For a gap distance in the 3-7 nm range, the CL spectral features of the dimer do not significantly depend on this parameter, as shown in Figure S5a. For a trimer, the asymmetric gap distances of 3 and 5 nm also do not significantly influence the spectral shapes (Figure S5b).

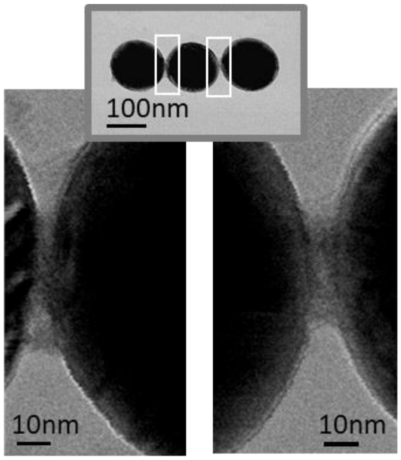

Figure S4. Magnified bright-field STEM images at the gap of the spheres in a trimer.

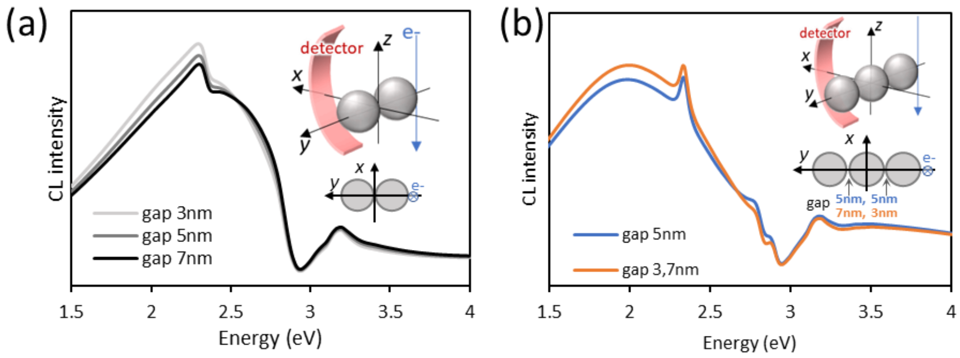

Figure S5. (a) CL spectra of a 120 nm Si dimer with different gap distances ranging from 3 to 7 nm calculated by using the MESME code. The rest of the condition is same as Figure 4a in the main text. (b) Calculated CL spectra of a trimer with different gap distances as shown in the inset.

S5. Contribution of ED modes in a dimer

Figure S6 compares CL spectra calculated by using MESME when all the multipolar modes are included or when only ED modes are retained. Clearly ED modes (including radial high-order modes at high energies) dominate the response of the system when the electron passes right next to the dimer.

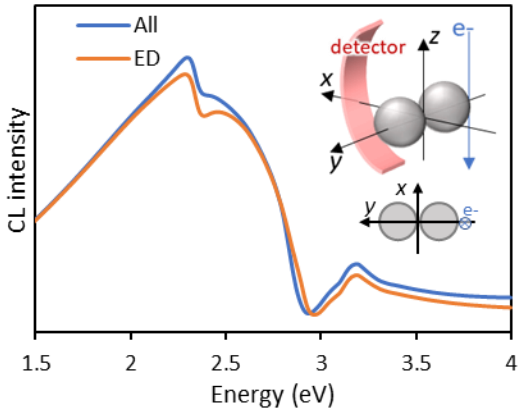

Figure S6. Comparison of CL spectra calculated with MESME when all the multipolar modes are included (blue) and when only ED modes are retained (orange). The rest of the condition is same as Figure 4a in the main text.
